# Plasma Metabolomic Analysis Reveals the Relationship between Immune Function and Metabolic Changes in Holstein Peripartum Dairy Cows

**DOI:** 10.3390/metabo12100953

**Published:** 2022-10-06

**Authors:** Zhuo Yang, Fang Luo, Guolin Liu, Zhengzhong Luo, Sijia Ma, Hang Gao, Hailong He, Jinzhong Tao

**Affiliations:** 1 Agriculture College, Ningxia University, Yinchuan 750021, China; 2 Department of Clinical Veterinary Medicine, College of Veterinary Medicine, Sichuan Agricultural University, Chengdu 611130, China

**Keywords:** dairy cattle, metabolism, immune function, tryptophan, transition period

## Abstract

Dairy cows undergo dynamic physiological changes from late gestation to early lactation, including metabolic changes and immune dysfunction. The aim of this study was to investigate the relationship between immune function and metabolic changes in peripartum dairy cows. Fifteen healthy Holstein dairy cows were enrolled 14 days prior to parturition, and plasma was collected on day −7, 0, 7, and 21 relative to calving. Plasma non-esterified fatty acids (NEFAs), glucose, β-hydroxybutyric acid (BHBA), immunoglobulin G (IgG), tumor necrosis factor alpha (TNF-α), and interleukin-2 levels were measured, and metabolic profiles were determined using ultra-high-performance liquid chromatography–quadrupole time-of-flight mass spectrometry. The data were analyzed using Tukey–Kramer adjustment for multiple comparisons, and multivariate and univariate statistical analyses were performed to screen for differential metabolites. The results showed that the concentrations of NEFAs, glucose, BHBA, and TNF-α in the plasma significantly increased and concentrations of IgG and interleukin-2 in plasma significantly decreased from −7 d to the calving day (*p* < 0.05). Additionally, the concentrations of glucose, IgG, and TNF-α significantly decreased from 0 to +7 d, and concentrations of NEFAs decreased significantly from +7 to +21 d (*p* < 0.05). The following six primary metabolic pathways were identified in all time point comparisons, and L-glutamate, linoleic acid, taurine, and L-tryptophan were involved in these major metabolic pathways. Correlation and pathway analyses indicated that a negative energy balance during the transition period adversely affects immune responses in cows, and L-tryptophan exerts immunomodulatory effects through the Trp-Kyn pathway, resulting in depletion of Trp and elevation of Kyn.

## 1. Introduction

The twenty-one days before and after calving in dairy cows are generally referred to as the transition period [1]. In this critical time, cows must adapt to a series of abrupt changes from pregnancy to calving and lactation [2]. During this period, cows experience a dramatic negative energy balance (NEB), because the energy intake does not match their energy requirements [3,4]. The dry matter intake of cows from third parity or after decreases by approximately 52% during the 14 days before calving [5,6], and 60–80% of their loss in body mass is attributed to loss of body fat [7,8]. As a consequence of fat mobilization, higher levels of non-esterified fatty acids (NEFAs) are produced in adipose tissue and released into blood [9]. Furthermore, the physiological challenge of fat mobilization can lead to excessive hepatic lipid peroxidation and produces numerous free radicals, resulting in decreased immune function [10]. For instance, according to Ospina et al., cows with blood concentrations of NEFAs > 0.29 mmol/L in the pre-partum period or >0.57 mmol/L in the post-partum period were more vulnerable to developing conditions such as retained placenta, ketosis, and displaced abomasum [11]. However, during the transition period, cows not only mobilize their body fat to overcome energy deficiencies, but also undergo dramatic alterations in other biological processes such as glucose and amino acid metabolism.

Changes in metabolic pathways and their significance for preserving health have been intensively studied in periparturient dairy cows. According to Reynolds et al., 10–30% of amino acids in peripartum dairy cows were converted into glucose through liver gluconeogenesis to relieve the NEB [12]. Luo et al. found that the levels of certain amino acids in the plasma of cows were significantly changed during parturition, including Val, Ile, Trp, Glu, and Arg [13]. Zhang et al. found that Ile, Leu, Lys, and Kyn were significantly increased in the serum of cows with ketosis [14]. However, amino acid metabolism in dairy cows during the peripartum period is not well understood.

Metabolomics is a tool for identifying the metabolite profiles of biological samples and for identifying biomarkers for diagnostics [15,16,17]. In recent years, this technology has been applied to the research on ruminants, and it can be used to better understand the changes in biological processes and relationships of metabolites in cows under specific conditions [18,19]. We hypothesized that changes in plasma metabolites are involved in regulating the immune function of dairy cows during the transition period. Based on previous data on the metabolome of cows, non-targeted metabolomic data were used to identify differential metabolites at −7, 0, +7, and +21 days relative to calving. We also examined immunoglobulin G (IgG), tumor necrosis factor (TNF)-α, and interleukin (IL)-2. The results showed that IgG is a central molecule of the mammalian immune system [20], TNF-α has an important role in the differentiation and regulation of immune cells [21], and IL-2 can induce proliferation of T cells and enhance the immune response function of T cells and B cells [22]. Therefore, we have chosen these indicators directly or indirectly reflecting the immune status of cows. This study was intended to investigate the relationship between immune function and metabolic changes in peripartum dairy cows. This study improves a better understanding for further studies on metabolic regulation in relation to the health of dairy cattle.

## 2. Materials and Methods

### 2.1. Animal Diet and Management

All procedures involving animals were performed according to the Care and Use of Laboratory Animal Guidelines for ethical review of animal welfare (approval number: GB/T 35892-2018). Fifty Holstein dairy cows were selected from Ningxia Dairy Farm (Ningxia, China). The cows had similar parity (3.20 ± 0.41), body condition scores (3.25 ± 0.49 (5-point system)), and calving days (280 ± 2.23 days). The cows were transferred to maternity stalls when they showed signs of calving and then transferred to a fresh pen after calving. Two pens had the same facilities and equipment on day 21 of the due date. All cows were under constant veterinary care and had free access to fresh water and were fed with total mixed rations.

### 2.2. Sample Collection and Preparation

According to the expected date of delivery (280 days of gestation), samples from the caudal vein were collected from 15 clinical healthy Holstein cows and added to 10 mL EDTA tubes at 9 a.m. on −7, 0, +7, and +21 d relative to calving. The plasma was obtained using heparin sodium as an anticoagulant, followed by centrifugation for 10 min (1500× *g*) within 1 h of collection. All samples were extracted into 2 mL Eppendorf tubes and stored at −80 °C for further analyses.

### 2.3. Plasma Analysis

The concentrations of NEFAs, glucose, IL-2, TNF-α, aspartate aminotransferase (AST), alkaline phosphatase (ALP), gamma-glutamyltransferase (GGT), and IgG in plasma were determined using commercial test kits purchased from Nanjing Jian Cheng Institute of Biological Engineering (Nanjing, China; #A042-2-1, #F006-1-1, #H003, #C010-2-1, #A059-2-2, #C017-2-1, #A052-1, #H106-1-1, respectively). All procedures were performed strictly in accordance with the manufacturer’s instructions.

### 2.4. Sample Metabolite Extraction

Blood plasma samples were thawed at 4 °C for 15 min; 100 μL blood from each sample was mixed with 400 μL extraction solvent (V methanol:V acetonitrile = 1:1) to remove proteins, followed by vortexing for 30 s. After storage at −20 °C for 60 min, the samples were centrifuged at 12,000 rpm for 15 min at 4 °C. The resulting supernatant was collected and analyzed. Quality control samples were prepared by mixing an equal aliquot of the supernatants to evaluate the system’s stability before testing.

### 2.5. Ultra-High-Performance Liquid Chromatography–Quadrupole Time-of-Flight Mass Spectrometry Analysis and Data Processing

Metabolic profiling of plasma was performed on a ultra-high-performance liquid chromatography system (1290, Agilent Technology, Santa Clara, CA, USA) coupled to a time-of-flight mass spectrometry (Agilent 6530; Agilent Technologies, Santa Clara, CA, USA) platform. We used a UPLC BEH Amide column (1.7 μm, 2.1 × 100 mm, Waters, Milford, MA, USA) and TripleTOF 6600 (AB Sciex, Framingham, MA, USA). The mobile phase consisted of 25 mM NH_4_OAc and 25 mM NH_4_OH in water (pH 9.75) (A) and acetonitrile (B), and the following elution gradient was used: 0 min, 95% B; 7 min, 65% B; 9 min, 40% B; 9.1 min, 95% B; 12 min, 95% B, at 0.5 mL per min. The samples were kept at 4 °C during the whole analysis, and the injection volume was 2 µL. During liquid chromatography–mass spectrometry (LC-MS), the Triple TOF mass spectrometer was used to obtain tandem mass spectrometry on the basis of information dependence. In this mode, the acquisition software (Analyst TF 1.7, AB Sciex) constantly evaluates the full scan measurement mass spectrum data, and collects and triggers the acquisition of these spectra according to the preselected standard. In each cycle, 12 precursor ions with intensity greater than 100 were selected to be fragmented at 30 V collision energy. The electrospray ionization source conditions were as follows: 60 psi ion source gas 1; 60 psi ion source gas 2; 35 psi curtain gas; 65 °C source temperature; and 5000 or −4000 V ion spray voltage floating in positive or negative modes, respectively.

### 2.6. Statistical Analysis

The data of plasma biochemical indicators were analyzed using SPSS software (version 21.0 for Windows, SPSS, Inc., Chicago, IL, USA). Tukey–Kramer adjustment was applied for multiple comparisons. Results are expressed as the mean ± standard error. Differences with *p* ≤ 0.05 were considered significant. Multi-dimensional statistical analysis of the ion peaks was performed using SIMCA-P 14.1 software (Umetrics, Umea, Sweden) and mainly included unsupervised principal component analysis and supervised orthogonal partial least squares discriminant analysis (OPLS-DA). The model was judged by parameters R^2^Y (model interpretation rate). When R^2^Y was >0.4 and the Q^2^ intercept was <0, the models were considered to be stable and reliable.

Univariate analysis was performed, and fold-change analysis was conducted to compare the relative abundance of metabolites between groups. A variable importance in projection value of >1 in OPLS-DA and *p* value of ≤0.05 in the *t*-test were used as screening criteria. Significantly different metabolites between different parturition time points were screened and used for metabolic pathway enrichment analysis of different time groups. Kyoto Encyclopedia of Genes and Genomes pathway analysis was performed using MetaboAnalyst 5.0 (http://www.metaboanalyst.ca accessed on 25 May 2021) software. Spearman rank correlation coefficients were calculated to examine the association of phenotypic variables and key differential metabolites using Origin software (OriginPro 2021; OriginLab Corporation, Northampton, MA, USA).

## 3. Results

### 3.1. Plasma Analyses

**Figure 1:**
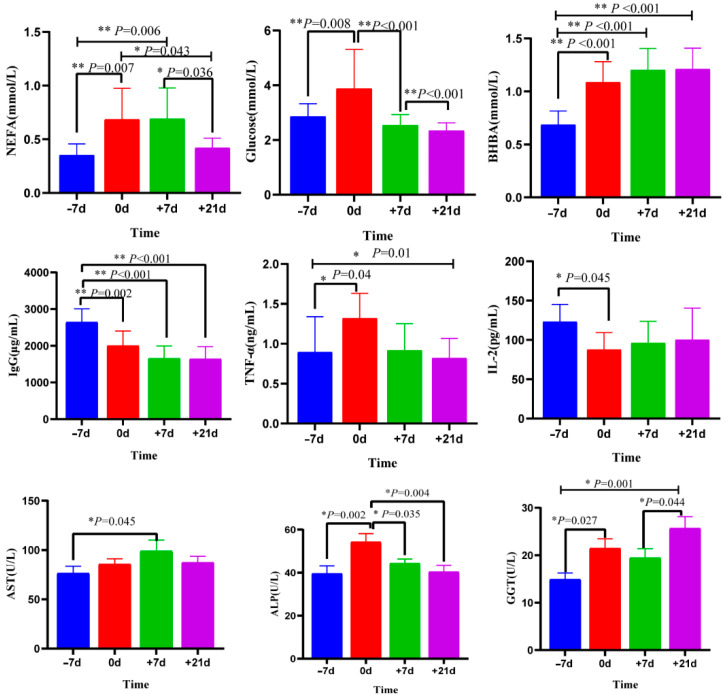
Plasma concentrations of non-esterified fatty acids (NEFAs), β-hydroxybutyric acid (BHBA), glucose, immunoglobulin G (IgG), tumor necrosis factor (TNF)-α, interleukin (IL)-2, AST, ALP, and GGT at −7, 0, +7, and +21 d. Data are expressed as the mean ± SE, * represent *p* < 0.05; ** represent *p* < 0.01.

The concentrations of NEFAs, β-hydroxybutyric acid (BHBA), glucose, IgG, TNF-α, IL-2, AST, ALP, and GGT in the plasma on day −7 before expected calving and on days 0, 7, and 21 relative to calving are presented in Figure 1. From −7 to 0 d, the concentrations of NEFAs, glucose, BHBA, TNF-α, ALP, and GGT in plasma increased by 97.14%, 35.78%, 58.60%, 52.16%, 37.17%, and 44.06%, respectively (*p* < 0.05), whilst the concentrations of IgG and IL-2 decreased by 24.16% and 31.01%, respectively (*p* < 0.01). The concentrations of glucose, IgG, and TNF-α on +7 d significantly decreased by 34.37%, 17.28%, 29.44%, and 18.34%, respectively, compared to 0 d (*p* < 0.05), while there were no significant differences in NEFAs, BHBA, and IL-2 levels during this period (*p* > 0.05). From +7 to +21 d, NEFAs significantly decreased by 39.13% and the concentrations of GGT increased by 32.01% (*p* < 0.05), without significant differences found in other indicators during this period (*p* > 0.05).

### 3.2. Metabolite Profile Analysis

**Figure 2:**
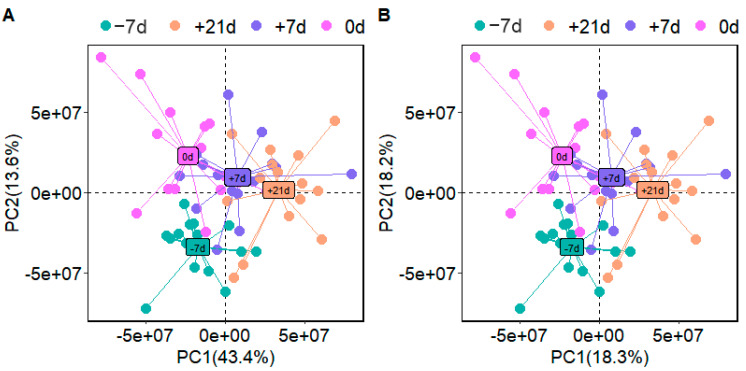
Principal component analysis score plot for −7, 0, +7, and +21 d samples analyzed in positive ion mode (**A**) and negative ion mode (**B**). PC1—first principal component; PC2—second principal component.

**Figure 3:**
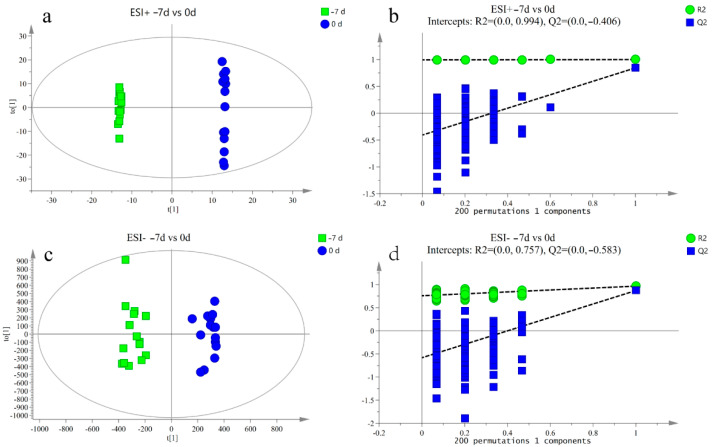
(**a**,**b**) Orthogonal partial least squares discriminant analysis (OPLS-DA) of scores and permutation test plots for the −7 d and 0 d group samples analyzed in the positive ion mode, respectively. (**c**,**d**) Orthogonal partial least squares discriminant analysis of scores and permutation test plots for the −7 d and 0 d group samples analyzed in the negative ion mode, respectively. t[1]—first principal component; to [1]—second orthogonal component. The intercept limit of Q^2^, calculated by regression line, is the plot of Q^2^ from permutation test in the OPLS-DA model.

**Figure 4:**
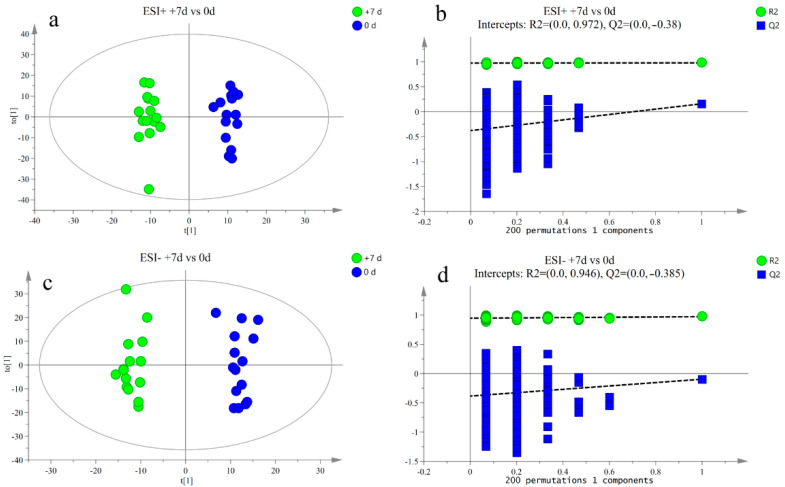
(**a**,**b**) Orthogonal partial least squares discriminant analysis (OPLS-DA) of scores and permutation test plots for the +7 d and 0 d group samples analyzed in the positive ion mode, respectively. (**c**,**d**) Orthogonal partial least squares discriminant analysis of scores and permutation test plots for the +7 d and 0 d group samples analyzed in the negative ion mode, respectively. t[1]—first principal component; to [1]—second orthogonal component. The intercept limit of Q^2^, calculated by regression line, is the plot of Q^2^ from permutation test in the OPLS-DA model.

**Figure 5:**
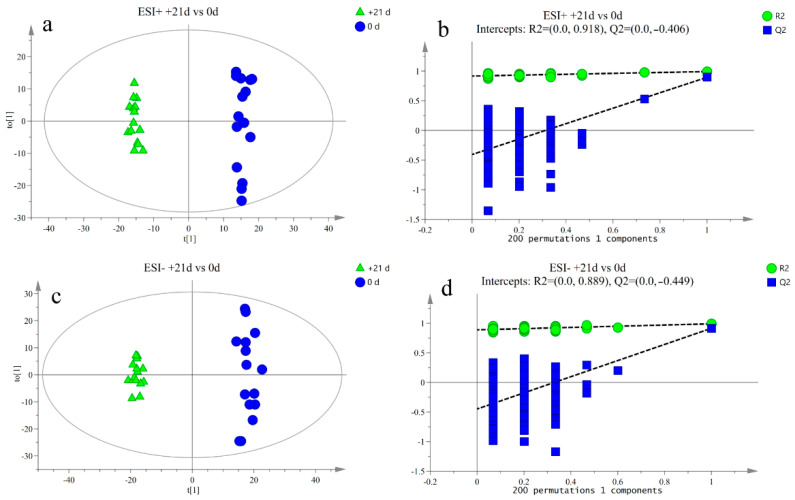
(**a**,**b**) Orthogonal partial least squares discriminant analysis (OPLS-DA) of scores and permutation test plots for the +21 d and 0 d group samples analyzed in the positive ion mode, respectively. (**c**,**d**) Orthogonal partial least squares discriminant analysis of scores and permutation test plots for the +21 d and 0 d group samples analyzed in the negative ion mode, respectively. t[1]—first principal component; to [1]—second orthogonal component. The intercept limit of Q^2^, calculated by regression line, is the plot of Q^2^ from permutation test in the OPLS-DA model.

**Figure 6:**
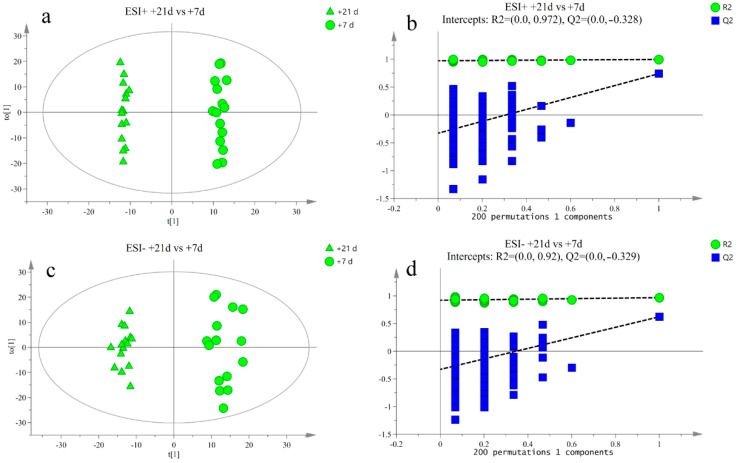
(**a**,**b**) Orthogonal partial least squares discriminant analysis (OPLS-DA) of scores and permutation test plots for the +21 d and +7 d group samples analyzed in the positive ion mode, respectively. (**c**,**d**) Orthogonal partial least squares discriminant analysis of scores and permutation test plots for the +21 d and +7 d group samples analyzed in the negative ion mode, respectively. t[1]—first principal component; to [1]—second orthogonal component. The intercept limit of Q^2^, calculated by regression line, is the plot of Q^2^ from permutation test in the OPLS-DA model.

The response intensity and retention time of each peak mostly overlapped, and the total ion chromatogram peak shape were intact. The response differences (RSD) of L-2-chlorophenylalanine in the QC samples in the positive and negative ion modes are shown in Supplementary Table S1. Adjacent peaks showed obvious separation, indicating that the method used was robust, stable, and highly repeatable. Thus, the chromatographic and mass spectrometric conditions were suitable for sample identification. After preprocessing the ion peak data, multidimensional statistical analysis was performed. The principal component analysis (PCA) score plot showed that the samples from different time points were well separated, and samples within the same groups were aggregated (Figure 2). To analyze the differences among the time points, longitudinal orthogonal partial least squares discriminant analysis (OPLS-DA) was performed. The OPLS-DA score plots and permutation score plots for each time point are shown in Figure 3, Figure 4, Figure 5 and Figure 6. The model parameters in positive and negative ion modes for R^2^Y are shown in Supplementary Table S2, where the test results were >0.4, indicating that the model was stable and reliable. Cross-validation and permutation tests for the model in positive and negative ion modes showed that the Q^2^ intercepts were <0 (Supplementary Table S2), indicating that the OPLS-DA model was not overfitted and that the metabolites differed at each time point.

### 3.3. Differential Metabolite Analysis

**Figure 7:**
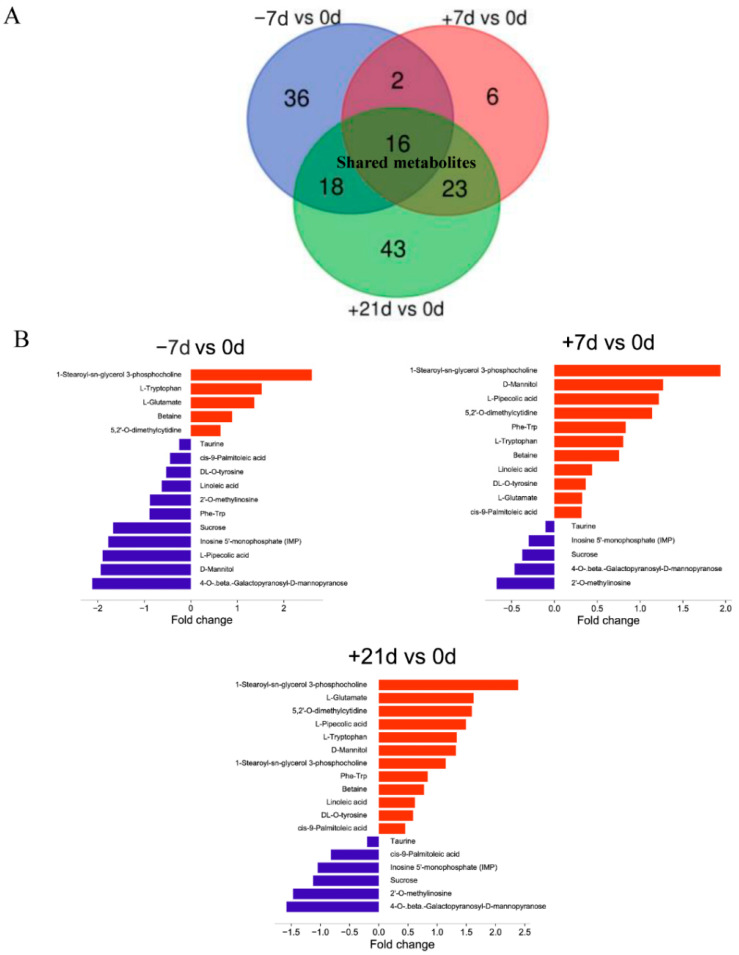
Comparisons of metabolite profiles between −7 vs. 0 d, +7 vs. 0 d, and +21 vs. 0 d. (**A**) Sixteen metabolites were shared among these three comparisons. (**B**) Bar plot showing the fold-change in shared metabolites in each comparison, where red and blue represent high and low levels, respectively, in the 0 d group.

The variable importance in projection was obtained from the *p*-value in the *t*-test and the OPLS-DA model. Based on a variable importance in projection > 1, *p* < 0.05, and fold-change threshold of >2 or <0.5, differential metabolites across the experimental groups were identified. Seventy-four differential metabolites were identified between −7 and 0 d (Supplementary Table S3). Fifty differential metabolites were identified on day +7 d compared to on 0 d (Supplementary Table S4). There were 106 differential metabolites between +21 and 0 d (Supplementary Table S5) and 82 differential metabolites between +21 and +7 d (Supplementary Table S6). There were 16 shared metabolites were identified in all time point comparisons (Figure 7): Phe-Trp, L-pipecolic acid, L-glutamate, inosine 5′-monophosphate, D-mannitol, DL-O-Tyr, betaine, 5,2′-O-dimethylcytidine, 4-O-beta-galactopyranosyl-D-mannopyranose, 2′-O-methylinosine, *cis*-9-palmitoleic acid, 1-stearoyl-*sn*-glycerol 3-phosphocholine, linoleic acid, L-Trp, sucrose, and taurine.

### 3.4. Metabolic Pathway Enrichment Analysis

**Figure 8:**
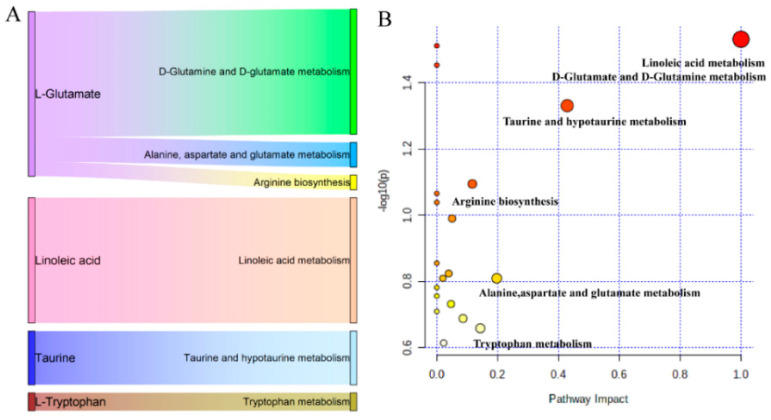
(**A**) Sankey plot showcasing key metabolites involved in significant enriched pathways. The dot plot shows the impact score and total number of metabolites in each enriched pathway (false discovery rate-adjusted *p* < 0.05). (**B**) Metabolic pathway analysis. *x*-axis, pathway impact; *y*-axis, −log10 (*p*). Circles represent the pathway impact score. Darker circles indicate more significant changes in metabolites in the corresponding pathway. The size of the circle corresponds to the metabolite counts in the corresponding pathway.

**Figure 9:**
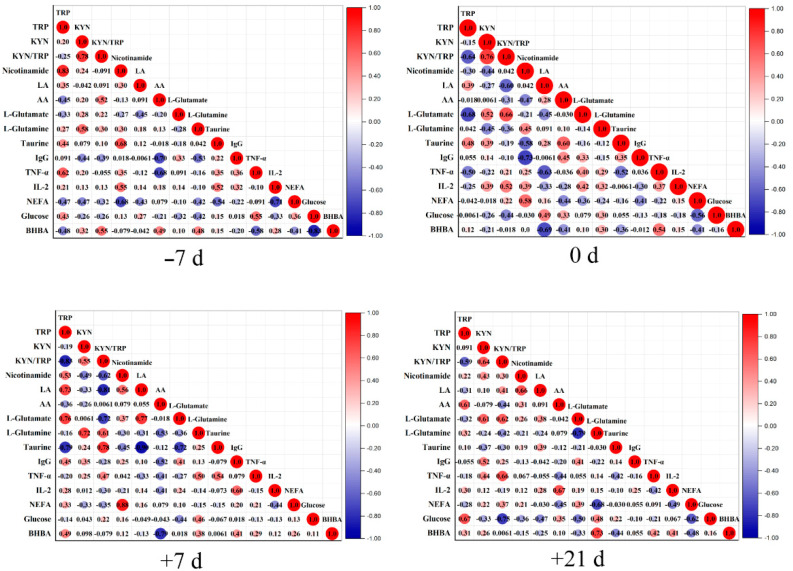
Spearman correlation heat maps for correlations between phenotypic variables (NEFAs, BHBA, IgG, IL-2, and TNF-α) and key differential metabolites on −7, 0, +7, and +21 d relative to calving. The scale indicates the level of correlations. KYN—kynurenine; TRP—tryptophan; LA—linoleic acid; AA—arachidonic acid; KYN/TRP—kynurenine-to-tryptophan ratio.

**Figure 10:**
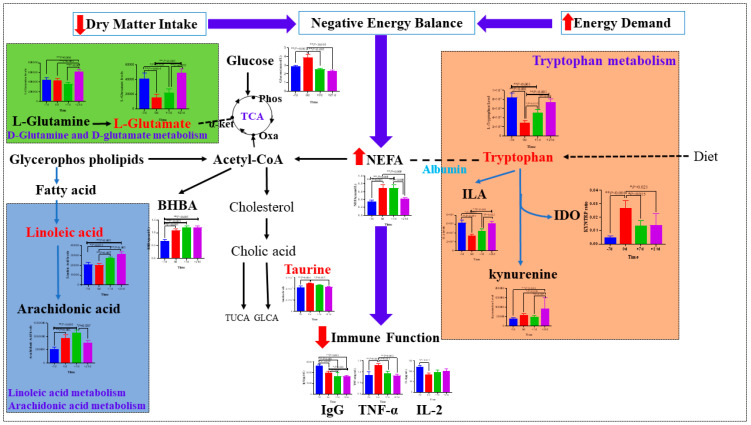
Metabolic pathways involving major metabolites with different plasma concentrations on −7, 0, +7, and +21 d relative to calving. (+) indicates increased level and (−) indicates decreased level. TCA—tricarboxylic acid; IDO—indoleamine 2,3-dioxygenase; TUCA—taurocholic acid; GLCA—glycocholic acid; Phos—phosphenol pyruvic acid; α-KET—α-ketoglutaric acid; Oxa—oxaloacetate.

To further analyze the dynamic changes in differential metabolites, MetaboAnalyst 5.0 was used to analyze metabolic pathway enrichment. A metabolic pathway with an impact value > 0.1 and *p* < 0.05 was regarded as the most relevant pathway. A total of six primary metabolic pathways were identified in all time point comparisons, including D-glutamate and D-glutamine metabolism; linoleic acid metabolism; taurine and hypo-taurine metabolism; alanine, aspartate, and glutamate metabolism; tryptophan metabolism; and Arg biosynthesis (Figure 8B). Only four critical different metabolites were involved in these six major metabolic pathways: L-glutamate, linoleic acid, taurine, and L-tryptophan (Figure 8A). The results from pathway analysis indicated that cows undergo substantial changes in metabolic responses during the transition period, mainly involving lipid and amino acid metabolism. The results of Spearman correlation analysis are shown in Figure 9. We subsequently queried metabolites that differed among groups in the Kyoto Encyclopedia of Genes and Genomes pathway database and searched published articles for data related to the overall metabolism pathway (Figure 10). 

## 4. Discussion

According to Reynolds et al., dairy cows require approximately 1 kg of glucose per day during late gestation; this demand increases to around 2.5 kg per day in the first 3 weeks postpartum [12]. Therefore, cows must mobilize their body fat reservoirs, which may lead to high levels of NEFAs being released into the blood [23]. NEFAs are a widely used indicator of body fat mobilization. Ospina et al. reported that the critical concentrations of NEFAs are approximately 0.29 mmol/L prepartum and 0.57 mM postpartum [11]. Moreover, body fat mobilization typically begins as early as several weeks before calving and intensifies during and after calving, as the net energy for lactation significantly increases postpartum [24]. In this study, the elevated NEFAs concentration from −7 d (0.35 mM) to +7 d (0.69 mM) indicates that the cows were suffered from an NEB.

Many studies have revealed relationships between an NEB and immune suppression of cows in the early days after caving [25,26]. In this study, indices related to immunity (IgG, TNF-α, and IL-2) in the plasma were measured to examine immune function in cows [27]. We first correlated the plasma NEFAs to these immune indicators to determine the general immune status of periparturient cows. The plasma TNF-α level increased as calving approached and remained low throughout early lactation, which is consistent with the results of previous studies [28,29]. The IL-2 level decreased before calving, which is similar to the results of Gao et al. [30]. The IgG concentrations decreased significantly prepartum, which is also consistent with the results of a previous study [31]. NEFAs were negatively correlated with IgG at −7 d, and 0 d and negatively correlated with IL-2 at −7 d, +7 d, and +21 d. Excessive NEFAs also can induce liver oxidative stress and lead to inflammatory cytokine in hepatocyte injury through the activation of nuclear factor kappa B (NF-κB) [32]. The major biomarkers of hepatocytic injury include elevated levels of intracellular enzymes such as AST, GGT, and ALP [33]. In this study, the concentrations of ALP and GGT in plasma were significantly increased before calving, indicating hepatocellular injury. These results demonstrate that a severe NEB during the transition period in cows adversely impacts immune function by reducing the expression of antibodies and other inflammatory cytokines.

An increasing number of studies have shown that amino acids play an important roles in modulating immune responses in dairy cows [34,35]. In this study, we found that amino acid metabolism of L-tryptophan (Trp), linoleic acid, L-glutamate, and taurine significantly changed from late pregnancy to early lactation. Experiments in humans and rats showed that the tryptophan–kynurenine pathway was related to balancing initiation and suppression of the immune response [36,37]. However, the effects of Trp metabolism on immunity in dairy cows has not been widely examined. Interestingly, we found that Trp is closely associated with the regulation of immune function. The main reasons for this effect are as follows: the indoleamine 2,3 dioxygenase is the rate-limiting enzyme in kynurenine (Kyn) production in the Trp degradation pathway [38], and the Kyn/Trp ratio in plasma is a key indicator of the regulation of indoleamine 2,3 dioxygenase activity [39]. The Kyn/Trp ratio increased from 0.49% on −7 d to 1.41% on +21 d with increasing Kyn levels and decreasing Trp levels (Figure 5). Moreover, Trp can be converted into indole-3-lactate, which significantly affects the modulation of immune function [40]. Increases in Kyn metabolites would reduce the production of indole-3-lactate and lower immune function. Indeed, the level of indole-3-lactate decreased in the prepartum stage (Figure 5), and the Kyn/Trp ratio was significantly positively correlated with TNF-α on +21 d and negatively correlated with IgG on −7 d and +7 d. Therefore, Trp is an effective immunomodulatory agent through the Trp-Kyn pathway that depletes Trp and elevates Kyn.

Excessive lipid mobilization is accompanied by changes in immune function and inflammation [41]. Linoleic acid belongs to the family of omega-6 polyunsaturated fatty acids and can be converted into arachidonic acid (AA) in the body. Previous studies have demonstrated that linoleic and arachidonic acid have immunoinhibitory activities [42]. For example, AA is upregulated in typical inflammatory cells, suggesting that high AA levels indicate inflammation in the body [43]. In this study, LA and AA significantly increased from −7 to +7 d, which is similar to the changes observed in previous studies [13,18]. A significant negative correlation between AA and IgG at −7 d was observed in this study. Therefore, we found negative relationships between elevated circulating NEFAs, LA, and AA and immune function.

L-glutamate is normally converted into glutamine through Gln synthetase [44]. Glutamine can enhance the body’s immunity, relieve oxidative stress, and provide energy by affecting the fumaric acid synthesis in the tricarboxylic acid cycle [45]. Doepel et al. reported that high levels of glutamine may reduce the degradation of intestinal glucose, and therefore relieve the NEB in the body [46]. Correlation analysis revealed a negative relationship between L-glutamine and NEFAs at +21 d. Combined with the changes in glucose concentrations in the plasma, our results suggest that high levels of L-glutamate relieve the NEB and strengthen immunity by reducing glucose degradation.

Taurine is an antioxidant sulfur-containing amino acid accounting for 60–80% of all free amino acids [47]. Taurine participates in immune function regulation via its anti-apoptosis activities, antioxidant stress effects, and regulation of mitochondrial function [48]. Marcinkiewicz et al. [49] showed that taurine improves immune function. However, in our study, as taurine concentrations increased, IgG and IL-2 levels significantly decreased and TNF-α significantly increased before calving. These inconsistent results may be explained as follows: taurine reduces apolipoprotein B100 and lipid secretion, which are essential structural components of very low-density lipoproteins [50]. The biological roles of very low-density lipoproteins are primarily related to triglyceride transport and eventually to cholesterol transport to the tissues. In this study, we observed a significant increase in taurine from −7 to 0 d (Figure 5). More triglyceride may be deposited in the liver because of reduced very low-density lipoproteins in the prepartum period. 

## 5. Conclusions

An NEB in the transition period adversely affects immune function in cows. Four critical metabolites were significantly changed at all time points: L-glutamate, linoleic acid, taurine, and L-tryptophan. These metabolites are associated with the regulation of immune function involved in D-glutamate and D-glutamine metabolism; linoleic acid metabolism; taurine and hypo-taurine metabolism; alanine, aspartate, and glutamate metabolism; tryptophan metabolism; and Arg biosynthesis. In particular, L-tryptophan exerts immunomodulatory effects through the Trp-Kyn pathway, resulting in depletion of Trp and elevation of Kyn. Further metabolomics studies are needed to obtain more detailed information on the metabolites and immune modulation of cows in the transition period.

## Data Availability

The data presented in this study are available in article and supplementary material.

## Supplementary Materials

The following supporting information can be downloaded at: https://www.mdpi.com/article/10.3390/metabo12100953/s1. Table S1. The response differences (RSD) of L-2-chlorophenylalanine in the QC samples in the positive (a) and negative (b) ion modes; Table S2. Parameters of orthogonal partial least squares discriminant analysis; Table S3. Differential metabolites identified at −7 d vs 0 d in the positive or negative mode; Table S4. Differential metabolites identified at day +7 vs day 0 in the positive or negative mode; Table S5. Differential metabolites identified at +21 d vs. 0 d in the positive or negative mode; Table S6. Differential metabolites identified at +21 d vs +7 d in the positive or negative mode.
