# Supplementary material for: Plasma Metabolomic Analysis Reveals the Relationship between Immune Function and Metabolic Changes in Holstein Peripartum Dairy Cows
Source: Metabolites. 2022 Oct 6;12(10):953. doi: 10.3390/metabo12100953 (PMC9611258; doi:10.3390/metabo12100953)
Supplement: Supplementary file 1 [file metabolites-12-00953-s001.zip › metabolites-1924982-supplementary.pdf]

metabolites

MDPI

Supplementary Material

1 Supplementary Tables

Supplementary Table S1. The response differences (RSD) of L-2-chlorophenylalanine in the QC samples in the positive (a) and negative (b) ion modes.

2-Chloro-L-phenylalanine	The Positive Ion Mode			The Negative Ion Mode		
	m/z	R.T.(s)	RSD_{QC}	m/z	R.T.(s)	RSD_{QC}
Samples	200.05	241.62	0.0094	198.03	236.17	0.264

Note: The response difference (RSD) of L-2-chlorophenylalanine in the samples were ≤ 30%, indicating good stability of the instrument data acquisition.

Supplementary Table S2. Parameters of orthogonal partial least squares discriminant analysis.

Group	Ion Mode	Parameter	
		R^2Y	Q^2 Intercept[c]
-7 d vs. 0 d	ESI+[a]	1.00	-0.41
+7 d vs. 0 d	ESI+	0.98	-0.38
+21 d vs. 0 d	ESI+	0.99	-0.41
+21 d vs. +7 d	ESI+	1.00	-0.33
-7 d vs. 0 d	ESI-[b]	0.97	-0.58
+7 d vs. 0 d	ESI-	0.97	-0.38
+21 d vs. 0 d	ESI-	0.99	-0.45
+21 d vs. +7 d	ESI-	0.97	-0.33

Note: [a] ESI^+, positive ion mode. [b]ESI^-, negative ion mode.[c] Q^2 intercept, permutation tests evaluate parameters.

Supplementary Table S3. Differential metabolites identified of -7d vs 0d in the positive or negative mode

No.	Ions Mode	Metabolite	VIP	FC	-Log (*p*)	m/z	RT (s)
1	ESI^+	Uridine	1.15	0.32	1.68	245.08	153.95
2	ESI^+	Sphingomyelin (d18:1/18:0)	1.55	0.62	2.96	794.60	130.02
3	ESI^+	S-Methyl-5'-thioadenosine	1.35	0.00	2.30	298.10	73.40
4	ESI^+	Ser-Gly	1.32	0.31	2.17	223.09	278.17
5	ESI^+	Ribothymidine	1.21	0.46	1.83	259.09	339.41
6	ESI^+	Pseudouridine	1.29	0.33	2.08	245.07	278.08
7	ESI^+	p-Hydroxyphenylacetic acid	1.59	0.38	3.10	135.05	141.06
8	ESI^+	Phosphoglycolic acid	1.28	1.21	2.03	217.06	261.41
9	ESI^+	Phe-Trp	1.37	0.54	2.34	352.17	141.83
10	ESI^+	Pantothenate	1.25	0.72	2.00	220.12	257.19
11	ESI^+	Palmitic acid	1.40	0.74	2.41	274.28	62.28
12	ESI^+	Nicotinate	1.50	0.29	2.77	124.04	208.56
13	ESI^+	N6-methyladenosine	1.07	0.68	1.46	282.12	298.11

No.	Ions Mode	Metabolite	VIP	FC	-Log (*p*)	m/z	RT (s)
14	ESI^+	Lys-Pro	1.52	1.51	2.84	261.19	212.88
15	ESI^+	L-Pipecolic acid	1.38	0.27	2.35	130.09	262.96
16	ESI^+	L-Glutamate	1.26	2.58	1.97	130.05	284.22
17	ESI^+	L-Citrulline	1.89	2.73	4.62	176.10	385.48
18	ESI^+	Inosine 5'-monophosphate (IMP)	2.42	0.29	10.31	349.05	387.45
19	ESI^+	Indole-3-butyric acid	1.16	0.30	1.70	186.09	292.80
20	ESI^+	Ile-Tyr	1.10	0.28	1.58	294.16	306.98
21	ESI^+	Glu-Pro	1.64	0.67	3.34	227.10	369.45
22	ESI^+	gamma-L-Glutamyl-L-glutamic acid	1.18	0.55	1.79	277.10	328.80
23	ESI^+	D-Mannitol	1.61	0.26	3.19	243.10	24.22
24	ESI^+	DL-O-tyrosine	1.29	0.69	2.09	146.06	290.17
25	ESI^+	DL-Indole-3-lactic acid	1.56	1.89	3.05	188.07	252.23
26	ESI^+	D-Lactose	1.15	0.56	1.69	360.14	386.54
27	ESI^+	Cyclohexylamine	1.02	1.32	1.36	160.13	375.01
28	ESI^+	Creatinine	1.78	0.69	4.08	114.07	40.27
29	ESI^+	Creatine	1.21	0.51	1.87	132.08	404.64
30	ESI^+	Betaine	1.94	1.85	4.99	118.09	289.34
31	ESI^+	beta-Hydroxybutyrate	1.64	0.80	3.32	146.08	268.91
32	ESI+	Anthranilic acid (Vitamin L1)	1.62	0.41	3.22	138.06	279.33
33	ESI^+	Altretamine	1.83	0.20	4.24	228.20	38.86
34	ESI^+	Albuterol	1.17	0.43	1.72	300.18	265.66
35	ESI^+	Adenosine	1.27	0.35	2.04	309.13	381.16
36	ESI^+	Adenine	1.60	0.53	3.20	136.06	149.97
37	ESI^+	Acetoacetic acid	1.73	2.76	3.74	163.06	387.73
38	ESI^+	7-Methylxanthine	1.49	0.45	2.77	166.05	40.06
39	ESI^+	5,2'-O-dimethylcytidine	1.29	1.56	2.08	272.12	123.50
40	ESI^+	4-Pyridoxic acid	1.54	0.47	2.96	184.06	40.01
41	ESI^+	4-O-.beta.-Galactopyranosyl-D-mannopyranose	2.37	0.23	9.41	343.13	387.70
42	ESI^+	4-Guanidinobutyric acid	1.07	0.76	1.50	146.09	310.16
43	ESI^+	3-Ureidopropionate	1.93	0.41	4.92	133.06	305.18
44	ESI^+	3-Methylhistidine	1.21	0.73	1.86	170.09	429.93
45	ESI^+	2'-O-methylinosine	1.39	0.54	2.42	283.10	143.31
46	ESI^+	2-Ethyl-4-hydroxy-5-methyl-3(2H)-furanone	1.21	0.75	1.85	160.10	298.25
47	ESI^+	2-Ethoxyethanol	1.25	1.94	1.96	151.10	48.84
48	ESI^+	1-Stearoyl-sn-glycerol 3-phosphocholine	1.59	6.07	3.17	1069.72	154.21
49	ESI^+	1-Stearoyl-2-hydroxy-sn-glycero-3-phosphocholine	1.54	1.71	2.93	546.36	179.98
50	ESI^+	1-O-Octadecyl-sn-glyceryl-3-phosphorylcholine	1.71	2.14	3.65	554.35	255.22
51	ESI^+	1-Myristoyl-sn-glycero-3-phosphocholine	1.77	2.07	3.95	468.31	173.81
52	ESI^+	(R)-3-Hydroxybutyric acid	1.14	0.20	1.67	168.06	142.81
53	ESI^+	(3-Carboxypropyl)trimethylammonium cation	1.53	1.43	2.87	146.12	367.20
54	ESI^-	(S)-2-Hydroxyglutarate	1.62	0.66	3.33	147.03	376.93

No.	Ions Mode	Metabolite	VIP	FC	-Log (*p*)	m/z	RT (s)
55	ESI^-	3-Methoxy-4-Hydroxyphenylglycol Sulfate	2.93	0.26	3.69	263.02	39.94
56	ESI^-	3-Phenylpropanoic acid	1.45	1.44	1.66	149.06	97.93
57	ESI^-	Acetylglycine	5.49	0.23	3.30	116.03	267.17
58	ESI^-	Arachidonic Acid (peroxide free)	1.09	0.54	2.40	303.23	38.02
59	ESI^-	cis-9-Palmitoleic acid	3.01	0.73	1.48	313.24	162.74
60	ESI^-	Dihydrouracil	1.04	0.68	3.13	130.06	73.14
61	ESI^-	Dihydroxyacetone	1.30	0.38	2.50	71.01	252.05
62	ESI^-	gamma-L-Glutamyl-L-phenylalanine	1.28	0.56	2.44	310.15	253.18
63	ESI^-	Indoxyl sulfate	2.50	0.73	2.18	212.00	26.81
64	ESI^-	L-Glutamate	2.74	1.61	3.09	146.05	381.09
65	ESI^-	Linoleic acid	1.64	0.65	3.18	279.23	38.95
66	ESI^-	L-Leucine	6.52	1.44	1.76	130.09	258.18
67	ESI^-	L-Malic acid	2.19	0.60	1.83	133.01	397.32
68	ESI^-	L-Tryptophan	4.72	2.87	4.88	203.08	250.14
69	ESI^-	myo-Inositol	1.19	0.74	2.44	179.06	378.60
70	ESI^-	Oleic acid	5.67	0.33	7.02	281.25	38.33
71	ESI^-	Pantothenate	1.83	0.80	1.30	218.10	257.00
72	ESI^-	Sucrose	2.77	0.31	8.96	401.13	386.43
73	ESI^-	Taurine	4.58	0.84	1.55	124.01	282.04
74	ESI^-	Uracil	1.22	0.66	2.91	111.02	151.85

Note: ESI+ was positive ion model, ESI^- was negative ion model. Log (*P*) indicated the logarithmic function of *P*-value based on 10. -Log10 (0.05) = 1.3; m/z = mass-to-charge ratio; RT = retention time; VIP = variable importance in projection. FC=fold change. a Fold change greater than 1 indicates relatively higher concentration in 0 d group, whereas a fold change of less than 1 indicates a concentration lower in 0 d group. The same below.

Supplementary Table S4. Differential metabolites identified of day +7 vs day 0 in the positive or negative mode.

No.	Ions Mode	Metabolite	VIP	FC	-Log (*p*)	m/z	RT (s)
1	ESI^+	1-Stearoyl-sn-glycerol 3-phosphocholine	1.85	3.83	2.85	1069.72	154.21
2	ESI^+	Tetrahydro-L-biopterin	1.95	2.78	3.05	500.28	174.67
3	ESI^+	D-Mannitol	2.04	2.41	3.42	243.10	24.22
4	ESI^+	Glycochenodeoxycholate	1.57	2.40	1.89	432.31	198.03
5	ESI^+	L-Pipecolic acid	1.95	2.33	3.13	130.09	262.96
6	ESI^+	His-Gln	1.40	2.31	1.53	266.13	283.57
7	ESI^+	5,2'-O-dimethylcytidine	1.71	2.20	2.45	272.12	123.50
8	ESI^+	Pro-Gly	1.49	2.16	1.72	173.09	306.91
9	ESI^+	Glycodeoxycholic acid	1.49	2.11	1.65	450.32	198.04
10	ESI^+	Pro-Ser	1.42	2.08	1.66	203.10	276.59
11	ESI^+	Glycolithocholic acid	1.45	2.03	1.59	434.32	198.28
12	ESI^+	Glycocholic acid	1.75	1.88	2.34	483.34	238.77
13	ESI^+	Phe-Trp	1.34	1.78	1.45	352.17	141.83
14	ESI^+	1-Palmitoyl-sn-glycero-3-phosphocholine	1.46	1.73	1.79	478.33	176.53

No.	Ions Mode	Metabolite	VIP	FC	-Log (*p*)	m/z	RT (s)
15	ESI+	Betaine	1.94	1.69	3.23	118.09	289.34
16	ESI+	Cytidine	1.59	1.65	2.07	487.18	230.08
17	ESI+	D-Pipecolinic acid	1.25	1.61	1.48	171.11	253.96
18	ESI+	Nicotinamide	1.76	1.55	2.82	123.06	59.78
19	ESI+	Lys-Ser	1.67	1.54	2.17	216.14	326.06
20	ESI+	5-Methylcytidine	1.70	1.42	2.49	258.11	198.51
21	ESI+	PC (16:0/16:0)	1.29	1.42	1.44	756.56	50.26
22	ESI+	Stearic acid	1.43	1.38	1.54	302.31	58.75
23	ESI+	Methoxyacetic acid	1.84	1.37	3.06	151.06	106.92
24	ESI+	Linoleic acid	2.11	1.36	3.62	263.24	41.43
25	ESI+	DL-O-tyrosine	1.31	1.29	1.48	146.06	290.17
26	ESI+	cis-9-Palmitoleic acid	1.61	1.24	2.35	277.22	38.46
27	ESI+	D-Proline	1.23	1.11	1.41	116.07	302.00
28	ESI+	Methyl acetoacetate	1.20	1.07	1.32	134.08	337.33
29	ESI+	Inosine 5'-monophosphate (IMP)	1.25	0.81	1.59	349.05	387.45
30	ESI+	4-O-.beta.-Galactopyranosyl-D-mannopyranose	1.35	0.72	1.70	343.13	387.70
31	ESI+	IS	1.75	0.70	2.42	200.05	241.62
32	ESI+	2-Thiocytidine	1.45	0.68	1.52	260.07	168.58
33	ESI+	2'-O-methylinosine	1.45	0.63	1.64	283.10	143.31
34	ESI+	D-Lactose	1.91	0.54	2.75	360.14	386.54
35	ESI+	Adenosine	1.63	0.39	1.96	309.13	381.16
36	ESI+	Arg-Tyr	1.46	0.36	1.61	337.17	449.38
37	ESI+	Tyr-Glu	1.77	0.27	2.27	371.15	302.32
38	ESI-	2'-Deoxy-D-ribose	1.42	2.07	1.63	193.07	302.14
39	ESI-	2-Thiocytidine	2.75	0.57	2.42	258.06	245.16
40	ESI-	Glucosamine	1.09	2.27	2.09	179.08	130.25
41	ESI-	IS	4.04	1.34	1.43	198.03	236.17
42	ESI-	L-Carnitine	3.92	2.48	2.42	142.09	128.15
43	ESI-	L-Glutamate	1.87	1.25	1.44	146.05	381.09
44	ESI-	L-Tryptophan	3.11	1.74	1.93	203.08	250.14
45	ESI-	N-Acetyl-L-aspartic acid	1.25	1.58	1.96	174.04	375.95
46	ESI-	Nicotinamide ribotide	1.41	0.52	2.65	333.06	419.20
47	ESI-	Salicyluric acid	2.09	2.21	2.55	194.05	135.28
48	ESI-	Stearic acid	1.89	2.03	1.39	283.26	38.71
49	ESI-	Sucrose	1.42	0.77	1.74	401.13	386.43
50	ESI-	Taurine	4.15	0.93	1.70	124.01	282.04

Supplementary Table S5. Differential metabolites identified of +21d vs 0d in the positive or negative mode.

NO.	Ions Mode	Metabolite	VIP	FC	-Log (*p*)	m/z	RT (s)
1	ESI+	1-Stearoyl-sn-glycerol 3-phosphocholine	1.65	5.23	4.77	1069.72	154.21
2	ESI+	Pro-Ser	1.53	5.16	3.97	203.10	276.59

NO.	Ions Mode	Metabolite	VIP	FC	-Log (*p*)	m/z	RT (s)
3	ESI^+	Tetrahydro-L-biopterin	1.80	4.83	6.16	500.28	174.67
4	ESI^+	Glycochenodeoxycholate	1.21	3.98	2.76	432.31	198.03
5	ESI^+	Pro-Gly	1.61	3.72	4.94	173.09	306.91
6	ESI^+	5-Methoxytryptamine	1.57	3.66	4.80	235.08	306.91
7	ESI^+	Glycodeoxycholic acid	1.13	3.56	2.43	450.32	198.04
8	ESI^+	3-Methoxybenzoic acid	1.56	3.26	4.09	175.04	141.59
9	ESI^+	Glycolithocholic acid	1.11	3.24	2.34	434.32	198.28
10	ESI^+	Pro-Val	1.69	3.20	5.43	232.17	311.66
11	ESI^+	L-Glutamate	1.56	3.08	4.52	130.05	284.22
12	ESI^+	1-Palmitoyl-sn-glycero-3-phosphocholine	1.80	3.07	6.25	478.33	176.53
13	ESI^+	5,2'-O-dimethylcytidine	1.96	3.02	7.93	272.12	123.50
14	ESI^+	3-Hydroxyisovaleric acid	1.33	2.86	3.23	254.16	245.16
15	ESI^+	L-Pipecolic acid	1.19	2.82	2.44	130.09	262.96
16	ESI^+	p-Hydroxyphenylacetic acid	1.44	2.71	3.39	135.05	141.06
17	ESI^+	Cytidine	1.67	2.70	5.12	487.18	230.08
18	ESI^+	His-Gln	1.18	2.58	2.37	266.13	283.57
19	ESI^+	D-Mannitol	1.56	2.50	4.72	243.10	24.22
20	ESI^+	D-Pipecolinic acid	1.46	2.36	3.71	171.11	253.96
21	ESI^+	5-Hydroxyindoleacetate	1.72	2.36	5.14	233.09	192.91
22	ESI^+	Pro-Phe	1.41	2.29	3.56	227.11	330.29
23	ESI^+	3-Hydroxybenzoate	1.44	2.28	3.33	180.07	110.96
24	ESI^+	L-Citrulline	1.44	2.22	3.44	176.10	385.48
25	ESI^+	N6-Acetyl-L-lysine	1.62	2.08	4.74	189.12	338.93
26	ESI^+	1-O-Octadecyl-sn-glyceryl-3-phosphorylcholine	1.16	2.05	2.16	554.35	255.22
27	ESI^+	Glycocholic acid	1.23	2.04	2.79	483.34	238.77
28	ESI^+	1-Myristoyl-sn-glycero-3-phosphocholine	1.73	2.00	5.22	468.31	173.81
29	ESI^+	His-Gly	1.09	1.95	2.27	213.10	307.44
30	ESI^+	Ribothymidine	1.12	1.91	2.13	259.09	339.41
31	ESI^+	5-Methylcytidine	1.84	1.91	6.75	258.11	198.51
32	ESI^+	DL-Indole-3-lactic acid	1.53	1.85	3.67	188.07	252.23
33	ESI^+	4-(4-Chlorophenyl)-4-hydroxypiperidine	1.21	1.80	2.36	211.07	105.85
34	ESI^+	Lys-Ser	1.33	1.79	3.14	216.14	326.06
35	ESI^+	Phe-Trp	1.04	1.79	1.87	352.17	141.83
36	ESI^+	Nicotinamide	1.64	1.73	4.79	123.06	59.78
37	ESI^+	Betaine	1.37	1.71	3.14	118.09	289.34
38	ESI^+	N-Acetyl-L-alanine	1.12	1.71	2.27	114.06	309.18
39	ESI^+	Hippuric acid	1.05	1.54	1.84	180.07	38.96
40	ESI^+	Linoleic acid	1.32	1.54	3.22	263.24	41.43
41	ESI^+	Methoxyacetic acid	1.61	1.50	4.36	151.06	106.92
42	ESI^+	DL-O-tyrosine	1.22	1.50	2.46	146.06	290.17
43	ESI^+	1-Stearoyl-2-hydroxy-sn-glycero-3-phosphocholine	1.16	1.46	2.07	546.36	179.98

NO.	Ions Mode	Metabolite	VIP	FC	-Log (*p*)	m/z	RT (s)
44	ESI^+	L-Glutamine	1.11	1.44	2.21	188.10	339.15
45	ESI^+	Cytosine	1.47	1.43	3.86	112.05	199.00
46	ESI^+	cis-9-Palmitoleic acid	1.27	1.37	2.71	277.22	38.46
47	ESI^+	16-Hydroxypalmitic acid	1.02	1.36	1.74	295.23	38.21
48	ESI^+	Deoxycytidine	1.29	1.36	2.90	228.10	199.02
49	ESI^+	L-Proline	1.03	1.34	2.10	231.13	306.39
50	ESI^+	N-Acetylglutamine	1.04	1.30	2.03	189.09	370.70
51	ESI^+	D-Proline	1.73	1.27	5.41	116.07	302.00
52	ESI^+	(3-Carboxypropyl)trimethylammonium cation	1.02	1.13	1.78	146.12	367.20
53	ESI^+	Choline	1.24	0.89	2.50	104.11	272.13
54	ESI^+	Glu-Pro	1.22	0.77	2.86	227.10	369.45
55	ESI^+	3-Methylhistidine	1.34	0.72	2.96	170.09	429.93
56	ESI^+	Allantoin	1.43	0.66	3.54	176.08	175.42
57	ESI^+	Phosphoglycolic acid	1.74	0.53	6.22	217.06	261.41
58	ESI^+	Sphingomyelin (d18:1/18:0)	1.64	0.52	5.21	794.60	130.02
59	ESI^+	Inosine 5'-monophosphate (IMP)	1.94	0.48	7.59	349.05	387.45
60	ESI^+	2-Thiocytidine	1.46	0.43	4.07	260.07	168.58
61	ESI^+	Thymine	1.02	0.41	1.90	168.08	327.76
62	ESI^+	2'-O-methylinosine	1.61	0.36	4.82	283.10	143.31
63	ESI^+	4-O-.beta.-Galactopyranosyl-D-mannopyranose	1.90	0.33	7.33	343.13	387.70
64	ESI^+	Altretamine	1.48	0.24	4.01	228.20	38.86
65	ESI^+	25-Hydroxycholesterol	1.45	0.06	3.40	447.31	141.45
66	ESI^-	L-Threonate	1.05	0.73	1.89	135.03	326.22
67	ESI^-	Dihydrouracil	1.05	0.79	2.00	130.06	73.14
68	ESI^-	Deoxycytidine	1.09	1.78	2.06	226.07	203.77
69	ESI^-	Thymidine	1.12	0.67	2.21	241.08	96.43
70	ESI^-	Creatine	1.05	1.33	2.38	261.13	333.71
71	ESI^-	Arachidic acid	1.06	1.68	2.38	311.29	36.71
72	ESI^-	D-Fructose	1.11	1.60	2.38	359.11	261.85
73	ESI^-	L-Gulonic gamma-lactone	1.14	2.29	2.45	177.03	76.09
74	ESI^-	D-Mannose	1.13	0.78	2.46	239.08	290.78
75	ESI^-	Prostaglandin A2	1.16	0.36	2.56	393.23	42.09
76	ESI^-	Palmitaldehyde	1.20	0.62	2.80	299.26	42.37
77	ESI^-	5-Hydroxymethyluracil	1.18	1.74	2.84	158.06	136.82
78	ESI^-	2'-Deoxy-D-ribose	1.24	2.55	2.85	193.07	302.14
79	ESI^-	N-Acetyl-L-aspartic acid	1.24	1.68	2.87	174.04	375.95
80	ESI^-	16-Hydroxypalmitic acid	1.23	0.53	2.94	271.23	42.19
81	ESI^-	cis-9-Palmitoleic acid	1.23	0.57	2.97	313.24	162.74
82	ESI^-	L-Arabinose	1.31	0.66	3.05	149.05	274.58
83	ESI^-	Hippuric acid	1.28	0.69	3.31	216.02	163.52
84	ESI^-	Pseudouridine	1.31	0.64	3.33	243.06	234.00

NO.	Ions Mode	Metabolite	VIP	FC	-Log (*p*)	m/z	RT (s)
85	ESI-	L-Carnitine	1.36	3.34	3.45	142.09	128.15
86	ESI-	alpha-Ketocaproic acid	1.29	1.86	3.69	129.06	137.49
87	ESI-	D-Lyxose	1.43	2.43	3.80	209.07	211.68
88	ESI-	Salicyluric acid	1.36	3.42	3.90	194.05	135.28
89	ESI-	S-Methyl-5'-thioadenosine	1.44	2.43	3.99	296.08	200.27
90	ESI-	2-Methyl-3-hydroxybutyric acid	1.40	1.70	4.16	117.06	228.36
91	ESI-	L-Arabinono-1,4-lactone	1.46	0.64	4.24	295.23	42.17
92	ESI-	Isovalerylglycine	1.49	2.58	4.37	158.08	200.56
93	ESI-	Benzoic acid	1.53	4.78	4.56	181.05	24.51
94	ESI-	Glucosamine	1.53	4.35	4.64	179.08	130.25
95	ESI-	L-Tryptophan	1.49	2.52	4.81	203.08	250.14
96	ESI-	2-Thiocytidine	1.58	0.35	4.95	258.06	245.16
97	ESI-	Nicotinamide ribotide	1.59	0.30	4.97	333.06	419.20
98	ESI-	Taurine	1.57	0.87	5.02	124.01	282.04
99	ESI-	Synephrine	1.61	5.51	5.34	148.08	185.35
100	ESI-	beta-Nicotinamide D-ribonucleotide	1.57	2.10	5.48	351.09	25.81
101	ESI-	1-Stearoyl-sn-glycerol 3-phosphocholine	1.66	2.21	6.07	582.38	211.11
102	ESI-	N1-Methyl-4-pyridone-3-carboxamide	1.66	0.47	6.19	211.17	42.22
103	ESI-	Glycerol 3-phosphate	1.72	1.62	6.38	171.01	375.16
104	ESI-	myo-Inositol	1.69	0.60	6.67	179.06	378.60
105	ESI-	Sucrose	1.77	0.46	7.02	401.13	386.43
106	ESI-	L-homocysteic acid	1.74	1.66	7.11	242.05	20.82

Supplementary Table S6. Differential metabolites identified of +21d vs +7d in the positive or negative mode.

No.	Ions Mode	Metabolite	VIP	FC	-log (p)	m/z	RT (s)
1	ESI+	Pro-Ser	1.56	2.49	2.41	203.10	276.59
2	ESI+	Pro-Val	1.84	2.34	3.44	232.17	311.66
3	ESI+	L-Glutamate	1.73	2.24	2.93	130.05	284.22
4	ESI+	3-Methoxybenzoic acid	1.65	2.05	2.72	175.04	141.59
5	ESI+	3-Hydroxykynurenine	1.37	1.97	1.90	225.09	277.48
6	ESI+	1-O-Octadecyl-sn-glyceryl-3-phosphorylcholine	1.40	1.94	1.91	554.35	255.22
7	ESI+	2'-Deoxyuridine	1.17	1.88	1.47	229.08	40.53
8	ESI+	5-Hydroxyindoleacetate	2.03	1.86	4.10	233.09	192.91
9	ESI+	3-Hydroxyisovaleric acid	1.39	1.82	1.92	254.16	245.16
10	ESI+	N6-Acetyl-L-lysine	1.97	1.78	3.96	189.12	338.93
11	ESI+	p-Hydroxyphenylacetic acid	1.33	1.77	1.84	135.05	141.06
12	ESI+	5-Methoxytryptamine	1.35	1.77	1.84	235.08	306.91
13	ESI+	1-Palmitoyl-sn-glycero-3-phosphocholine	1.73	1.77	3.06	478.33	176.53
14	ESI+	L-Citrulline	1.51	1.77	2.20	176.10	385.48
15	ESI+	Tetrahydro-L-biopterin	1.43	1.74	2.01	500.28	174.67

No.	Ions Mode	Metabolite	VIP	FC	-log (p)	m/z	RT (s)
16	ESI^+	L-Glutamine	2.01	1.73	4.06	188.10	339.15
17	ESI^+	Pro-Gly	1.38	1.72	1.93	173.09	306.91
18	ESI^+	3-Hydroxybenzoate	1.60	1.71	2.52	180.07	110.96
19	ESI^+	Hippuric acid	1.64	1.71	2.63	180.07	38.96
20	ESI^+	Ribothymidine	1.43	1.67	2.02	259.09	339.41
21	ESI^+	1-Myristoyl-sn-glycero-3-phosphocholine	1.81	1.64	3.26	468.31	173.81
22	ESI^+	Cytidine	1.47	1.64	2.12	487.18	230.08
23	ESI^+	N-Acetyl-L-alanine	1.28	1.60	1.70	114.06	309.18
24	ESI^+	1-Oleoyl-sn-glycero-3-phosphocholine	1.14	1.60	1.34	522.36	166.22
25	ESI^+	1-Stearoyl-2-hydroxy-sn-glycero-3-phosphocholine	1.72	1.58	2.83	546.36	179.98
26	ESI^+	Pro-Phe	1.34	1.56	1.81	227.11	330.29
27	ESI^+	D-Pipecolinic acid	1.09	1.47	1.39	171.11	253.96
28	ESI^+	DL-Indole-3-lactic acid	1.31	1.41	1.70	188.07	252.23
29	ESI^+	5-Methylcytidine	1.52	1.35	2.30	258.11	198.51
30	ESI^+	His-Ala	1.34	1.34	1.73	268.14	403.75
31	ESI^+	Cytosine	1.91	1.31	3.70	112.05	199.00
32	ESI^+	L-Proline	1.39	1.27	2.03	231.13	306.39
33	ESI^+	Deoxycytidine	1.65	1.26	2.68	228.10	199.02
34	ESI^+	N-Acetylglutamine	1.17	1.23	1.41	189.09	370.70
35	ESI^+	beta-Hydroxybutyrate	1.16	1.17	1.37	146.08	268.91
36	ESI^+	D-Proline	1.53	1.15	2.31	116.07	302.00
37	ESI^+	Tyramine	1.32	0.87	1.74	120.08	248.14
38	ESI^+	L-Arabinose	1.23	0.86	1.52	168.09	248.25
39	ESI^+	Glu-Pro	1.28	0.80	1.61	227.10	369.45
40	ESI^+	3-Methylhistidine	1.67	0.74	2.76	170.09	429.93
41	ESI^+	3'-O-methyladenosine	1.64	0.73	2.65	282.12	124.23
42	ESI^+	Allantoin	1.56	0.73	2.37	176.08	175.42
43	ESI^+	Phenylacetylglycine	1.27	0.72	1.71	211.11	79.67
44	ESI^+	Thymine	1.16	0.71	1.48	168.08	327.76
45	ESI^+	2-Thiocytidine	1.21	0.63	1.60	260.07	168.58
46	ESI^+	Sphingomyelin (d18:1/18:0)	1.89	0.62	3.69	794.60	130.02
47	ESI^+	Chenodeoxycholate	1.46	0.61	2.04	410.34	34.65
48	ESI^+	Inosine 5'-monophosphate (IMP)	1.69	0.60	2.80	349.05	387.45
49	ESI^+	2'-O-methylinosine	1.25	0.58	1.60	283.10	143.31
50	ESI^+	Phosphoglycolic acid	2.13	0.58	4.85	217.06	261.41
51	ESI^+	L-Threonine	1.18	0.55	1.37	120.07	331.01
52	ESI^+	4-O-.beta.-Galactopyranosyl-D-mannopyranose	1.69	0.46	2.78	343.13	387.70
53	ESI^+	Altretamine	2.13	0.27	4.60	228.20	38.86
54	ESI^+	25-Hydroxycholesterol	1.73	0.12	2.76	447.31	141.45
55	ESI^-	Taurine	1.36	0.93	1.54	124.01	282.04
56	ESI^-	Synephrine	1.33	1.82	1.80	148.08	185.35

No.	Ions Mode	Metabolite	VIP	FC	-log (p)	m/z	RT (s)
57	ESI-	Sucrose	1.70	0.59	2.85	401.13	386.43
58	ESI-	Prostaglandin A2	1.65	0.41	3.06	393.23	42.09
59	ESI-	Palmitaldehyde	1.62	0.56	3.32	299.26	42.37
60	ESI-	Oleic acid	1.34	0.79	1.87	281.25	38.33
61	ESI-	Nicotinamide ribotide	1.71	0.58	2.93	333.06	419.20
62	ESI-	N1-Methyl-4-pyridone-3-carboxamide	1.53	0.63	2.56	211.17	42.22
63	ESI-	myo-Inositol	2.08	0.68	4.00	179.06	378.60
64	ESI-	L-Tryptophan	1.19	1.42	1.39	203.08	250.14
65	ESI-	L-Leucine	1.88	1.45	3.21	130.09	258.18
66	ESI-	L-Arabinono-1,4-lactone	1.59	0.58	3.10	295.23	42.17
67	ESI-	Isovalerylglycine	1.32	1.50	2.00	158.08	200.56
68	ESI-	Glycerol 3-phosphate	1.74	1.33	2.85	171.01	375.16
69	ESI-	Glycerol	1.54	0.75	2.14	151.06	231.32
70	ESI-	Glucosamine	1.36	1.86	1.86	179.08	130.25
71	ESI-	D-Mannose	1.29	0.84	1.61	239.08	290.78
72	ESI-	beta-Nicotinamide D-ribonucleotide	1.48	1.41	2.33	351.09	25.81
73	ESI-	Benzoic acid	1.19	1.89	1.49	181.05	24.51
74	ESI-	Arachidonic Acid (peroxide free)	1.31	0.70	1.92	303.23	38.02
75	ESI-	alpha-Ketocaproic acid	1.66	1.62	3.20	129.06	137.49
76	ESI-	Acetylglycine	1.32	0.59	1.87	116.03	267.17
77	ESI-	3-Methoxy-4-Hydroxyphenylglycol Sulfate	1.54	0.47	2.52	263.02	39.94
78	ESI-	3-Hydroxydodecanoic acid	1.23	0.62	1.92	215.16	43.21
79	ESI-	2-Thiocytidine	1.37	0.63	1.88	258.06	245.16
80	ESI-	2-Methyl-3-hydroxybutyric acid	1.19	1.30	1.48	117.06	228.36
81	ESI-	1-Stearoyl-sn-glycerol 3-phosphocholine	1.90	1.70	3.22	582.38	211.11
82	ESI-	16-Hydroxypalmitic acid	1.72	0.62	3.47	271.23	42.19
